# Alcohol and suicide in the Nenets Autonomous Okrug and Arkhangelsk Oblast, Russia

**DOI:** 10.3402/ijch.v75.30965

**Published:** 2016-07-22

**Authors:** Yury A. Sumarokov, Tormod Brenn, Alexander V. Kudryavtsev, Oleg Sidorenkov, Odd Nilssen

**Affiliations:** 1 Department of Community Medicine, UiT-The Arctic University of Norway, Tromsø, Norway; 2 International School of Public Health, Northern State Medical University, Arkhangelsk, Russia

**Keywords:** suicide, ethnicity, alcohol, indigenous, non-indigenous, distal risk factors, proximal risk factors

## Abstract

**Background:**

High suicide rates in the Russian North are coupled with high alcohol consumption in the described populations.

**Objective:**

To investigate the potential role of alcohol consumption on suicides in the Nenets Autonomous Okrug (NAO) in 2002–2012 and to compare this information with corresponding data from the neighboring Arkhangelsk Oblast (AO).

**Design:**

Retrospective population-based mortality study.

**Methods:**

Data from autopsy reports were used to identify 252 cases of suicide in the NAO and 1,198 cases in the AO in the period 2002–2012. Postmortem blood alcohol content (BAC) was available for 228 cases in the NAO and 1,185 cases in the AO. BAC as well as other selected variables were compared between the NAO and the AO among women and men, different age groups, ethnic groups, and selected variables of suicide.

**Results:**

Alcohol was present in the blood of 74.1% of male and 82.9% of female suicide cases in the NAO, which was significantly higher than the proportions found in the AO (59.3% of male and 46.6% female cases). BAC<1.0‰ and between 2.0 and 3.0‰ were more frequently found among suicide cases in the NAO than those in the AO.

**Conclusions:**

Our findings specify that alcohol drinking may be an essential risk factor for suicide in the NAO, and that this factor may be of greater importance in the indigenous population of the NAO than among Russians in the AO.

There is worldwide evidence that people who suffer from depression have a higher prevalence of suicide (1). Indeed, depression is recognized as the leading independent risk factor for suicide. Other important risk factors are alcohol and drug dependence, chronic diseases, including serious psychiatric disease, negative life events like acute changes in social and human relationships, economic problems, and fear of facing the consequences of one's actions when these actions are seen as shameful or regrettable.

Alcohol dependence and intoxication have been described as independent and powerful risk factors for suicide. Hufford (2) described the potential relationship between alcohol and suicide in two ways: a distal risk, defined as alcohol dependence over time, and a proximal risk, which is defined as the transformation of the statistical potential for suicide into action. As such, the latter relationship is linked to the timing of suicidal behavior. The mechanisms by which alcohol leads to suicide have been suggested as: (a) an increase in psychological distress, (b) an increase in aggressiveness, (c) driving suicidal ideations to action, and (d) constricted cognition impairing the generation and implementation of alternative coping strategies (2).

Reported proportions of alcohol use prior to suicide have ranged from 10% in South Africa to 69% in Finland (3). Studies from Eastern Europe found alcohol in the blood of 47.9% of suicide cases in Estonia (4), 62% in Belarus (5), and 60.2% in Russia (6). Other studies on suicides in Native Americans (7–10), the Sami in Sweden (11), Indigenous Canadians (12), and Australian Aborigines (13) have shown similar findings. Indeed, an overwhelming number of suicides have been reported among native populations in Canada, the USA, Norway, Sweden, Australia, New Zealand, Greenland, Brazil, and Russia (14). A common finding from all of these studies was the high alcohol consumption in the described populations.

Alcohol was introduced in the indigenous populations during their colonial history. Natively, it has never been a part of their traditional cultures. For example, Russian merchants of 19th–20th centuries played an important role in the drinking habits of the Nenets. The Nenets Autonomous Okrug (NAO) is located in the Russian North and is home to an indigenous people called the Nenets, who make up 17.8% of the population there. Previous papers have reported that the prevalence of suicides in the NAO is considerably higher than that found in other Russian regions and in Russia as a whole (14). Regional health workers have suggested that alcohol use may be the main risk factor for high suicide rates.

The aim of this study was to pursue local health workers’ hypothesis and investigate the postmortem presence of alcohol in the blood and the postmortem blood alcohol content (BAC) among suicide cases in the NAO. Furthermore, we compared our findings in the NAO with corresponding findings in the Arkhangelsk Oblast (AO), which is mostly populated by ethnic Russians.

## Materials and methods

### Study design

The present retrospective population-based mortality study included all autopsied suicide cases in the NAO and the AO that occurred from 1 January 2002 to 31 December 2012 (14). Each case of suicide was selected according to the definition as the act of deliberately killing oneself (1).

### Study site and population

The territory of NAO is situated in Northwest Russia, close to the Arctic Ocean. According to National Census data, in 2010 the total population of the NAO was 42,090 (15). The main ethnic group in the NAO is indigenous Nenets; non-indigenous groups include the Russians, Komi, and others. The Nenets are one of the largest indigenous population groups in Russia, and they have a large environmental, cultural, and social impact on the region. Many of them still follow the traditional lifestyle of nomadic or semi-nomadic reindeer herders and live in temporary (seasonal) settlements. The AO borders the NAO and covers a large part of Northwest Russia. The AO is one of the most ethnically Russian areas in the world: the proportion of Russians was 95.6% in 2010, compared to a proportion of indigenous Nenets of only 0.6% (15).

### Data sources and description

During the study period, 252 cases of suicide were registered in the NAO (215 males and 37 females). All cases had undergone forensic autopsy and had been classified in accordance with the *International Classification of Diseases*, 10th Revision (codes X60–X84 and Y87.0). All data from the NAO were collected by the first author (YS). Local health workers contributed to the work, and data from the regional forensic bureau were made available for this work. Twenty-four cases had missing or incomplete data on BAC and were excluded from further analyses. Correctness of the data from the different sources (except for laboratory data) was checked by local experts and re-checked by first author (YS).

During the same period, 1,185 cases of suicide were found in the database of death certificates in the AO. Autopsy records of these cases were reviewed to obtain data on variables that were not present in death certificates. Data in the AO were collected in the regional forensic bureau in Arkhangelsk, and the correctness of the data was checked by local experts and re-checked by one of the co-authors (OS). Thirteen cases had missing values for BAC, and were excluded from the analysis.

Variables of interest for which data were collected for this paper were presence of alcohol in the blood (BAC+), BAC, age, gender, ethnicity, and suicide method. BAC was measured by gas chromatography from blood taken at the autopsies (16).

### Data analyses

Alcohol consumption in the studied areas was estimated as liters of pure alcohol sold per year divided by the number of individuals over 15 years of age in the population (17). The chi-square test was used to identify non-random differences in the presence of alcohol in the blood and BAC between the studied groups. For small counts Fisher's exact test was used. Microsoft Excel and SPSS 22.0 were used for data storage and analysis.

### Ethical considerations

The study was approved on 23 June 2010 by the Ethical Committee of the Northern State Medical University, Arkhangelsk, Russia.

## Results

Complete data were available for 228 cases from our NAO dataset. Mean age for males was 36.0 years and 39.1 for females. Mean BAC was 2.02‰ in males and 2.14‰ in females that had alcohol in their blood at the time of autopsy. Complete autopsy data were available for 1,185 suicide cases (977 males and 208 females) from the AO. Mean age was 44.54 for males and 50.21 for females. Mean BAC was 2.54 and 2.56‰ in males and females, respectively.

### Presence of alcohol

In the NAO, both genders had a substantially higher proportion of BAC+ suicides than was seen in the AO (Tables I and II). Alcohol was present in the blood of 74.1% of male and 82.9% of female suicide cases in the NAO. Among the indigenous population, this proportion was even higher, with 78.3% and 92.3% in males and females, respectively. Data from the AO showed significantly lower proportions of BAC+ suicides, with 59.3% in males (Table I) and 46.6% in females (Table II).

**Table:** *Table I*. Number of suicides and proportion of cases with presence of alcohol in the blood (BAC+) in males by age in the Arkhangelsk Oblast (AO) and the Nenets Autonomous Okrug (NAO), 2002–2012

	AO		NAO		
			
Males Age (years)	Number of suicides	BAC+	Number of suicides	BAC+	p
10–19	53	27 (50.9%)	17	13 (76.5%)	0.038
20–29	155	98 (63.2%)	63	47 (74.6%)	0.000
30–39	174	118 (67.8%)	42	32 (76.2%)	0.598
40–49	230	151 (65.6%)	34	27 (79.4%)	0.074
50–59	181	106 (58.6%)	23	19 (82.6%)	0.155
60–69	102	54 (52.9%)	11	5 (45.5%)	0.023
≥70	82	25 (30.5%)	3	0 (0.0%)	0.011
Total	977	579 (59.3%)	193	143 (74.1%)	

BAC: blood alcohol content.

**Table:** *Table II*. Number of suicides and proportion of cases with presence of alcohol in the blood (BAC+) in females by age in the Arkhangelsk Oblast (AO) and the Nenets Autonomous Okrug (NAO), 2002–2012

	AO		NAO		
			
Females Age (years)	Number of suicides	BAC+	Number of suicides	BAC+	p
10–19	8	6 (75.0%)	4	4 (100%)	0.237
20–29	22	12 (54.5%)	6	5 (83.3%)	0.539
30–39	32	21 (65.6%)	12	11 (91.7%)	0.077
40–49	47	29 (61.7%)	8	7 (87.5%)	0.547
50–59	35	15 (42.8%)	2	1 (50.0%)	0.116
60–69	19	7 (36.8%)	0	0	0.351
≥70	45	7 (15.6%)	3	1 (33.3%)	0.681
Total	208	97 (46.6%)	35	29 (82.9%)	

BAC: blood alcohol content.

When stratified by age and gender, males in the NAO showed a higher percentage of BAC+ suicides than was found in the AO, especially in the age groups 10–19 and 20–29 years, whereas a lower percentage of BAC+ suicides was found in the age group 60–69 years. Higher proportions of BAC+ suicide cases were found among females aged >20 years in the NAO, compared with corresponding figures from the AO (Table II), but the difference did not reach statistical significance. Consequently, suicide cases that did not have any alcohol in their blood (BAC-) were more frequently seen in the AO than the NAO (Table III).

**Table:** *Table III*. Number of suicides by gender and blood alcohol content (BAC) in the Arkhangelsk Oblast (AO) and the Nenets Autonomous Okrug (NAO), 2002–2012

	AO		NAO			
				
BAC (‰)	Males	Females	Males	Females	p males	p females
0.0	398 (40.7%)	111 (53.4%)	47 (24.3%)	6 (17.1%)	0.000	0.000
>1.0	96 (9.8%)	12 (5.8%)	32 (16.6%)	6 (17.1%)	0.006	0.017
≥1.0–2.0	123 (12.6%)	21 (10.1%)	33 (17.1%)	4 (11.4%)	0.092	0.766
≥2.0–3.0	173 (17.7%)	34 (16.3%)	50 (25.9%)	17 (48.5%)	0.008	0.000
≥3.0	187 (19.2%)	30 (14.4%)	31 (16.1%)	2 (5.7%)	0.315	0.276
Total	977	208 (100%)	193 (100%)	35 (100%)		

### Blood alcohol content

BAC+ suicides were categorized into four BAC groups (Table IV). Both genders in the NAO more frequently displayed a BAC<1.0‰ or 2.0–3.0‰; p<0.0005). There was an equal number of suicide cases with a BAC>3.0‰ in both the NAO and the AO, and in both in males and females (Table III).

**Table:** *Table IV*. Distribution of suicide cases with and without alcohol present in the blood (BAC+/BAC−) by suicide method in the Arkhangelsk Oblast (AO) and the Nenets Autonomous Okrug (NAO), 2002–2012

	AO (N=1,185)		NAO (N=228)		
			
Suicide method	BAC+	BAC−	BAC+	BAC−	p
Hanging	626 (58.9%)	436 (41.1%)	126 (77.3%)	37 (22.7%)	0.000
Firearm use	19 (61.3%)	12 (38.7%)	34 (79.1%)	9 (20.9%)	0.094
Cutting	9 (29.0%)	22 (71.0%)	7 (46.7%)	8 (53.3%)	0.239
Other	22 (36.1%)	39 (63.9%)	5 (71.4%)	2 (28.6%)	0.104
Total	676 (57.0%)	509 (43.0%)	172 (75.4%)	56 (24.6%)	0.000

BAC: blood alcohol content.

### Alcohol and suicide methods

When comparing different suicide methods, BAC+ suicide cases commonly used the most violent suicide methods. BAC+ suicides by hanging or firearm use were seen more frequently in the NAO than in the AO (p=0.000) (Table IV). BAC+ suicides by hanging and cutting in males were significantly more common in the NAO than in the AO, and BAC+ suicides by hanging in females were also more frequent in the NAO than in the AO (Table V).

**Table:** *Table V*. Distribution of suicide cases with alcohol present in the blood (BAC+) by gender and suicide method in the Arkhangelsk Oblast (AO) and the Nenets Autonomous Okrug (NAO), 2002–2012

	AO (N=1185)		NAO (N=228)			
				
Suicide method	Males	Females	Males	Females	Males	Females
Hanging	534/879 (60.8%)	92/183 (50.3%)	102/133 (76.7%)	24/30 (80.0%)	0.000	0.002
Firearm use	19/31 (61.3%)	0/0 (0%)	31/40 (71.5%)	3/3 (100%)	0.137	0.111
Cutting	9/28 (32.1%)	0/3 (0%)	5/13 (38.5%)	2/2 (100%)	0.015	0.052
Other	17/39 (43.0%)	5/22 (22.0%)	5/7 (71.0%)	0/0 (0%)	0.174	0.588
Total	579/977 (59.3%)	97/208 (46.6%)	143/193 (74.1%)	29/35 (82.9%)	0.000	0.000

BAC: blood alcohol content.

### Other analyses

Analyses of socioeconomic characteristics (employment, education, urban–rural residence, and civil status) of the indigenous and non-indigenous populations of the NAO showed no difference in the proportion of BAC+ suicide cases.

## Discussion

To our knowledge, this is the first study to describe the connection between suicide and alcohol use in two neighboring regions of Russia with ethnically different populations. This paper considers the NAO and the AO as indigenous and non-indigenous areas, respectively, as the NAO comprises one of the largest indigenous populations in Russia, and 99% of the population of the AO is ethnically Russian. It is well known that suicide rates in Russia are among the highest in the world (18). In addition to South Siberia and the Far East, six Russian regions were mentioned as regions with the highest suicide rates in 2012: Altay, the NAO, Buryatia, the Jewish Autonomous Oblast, Chukotka, and Tyva (19). All of these regions contain high proportions of indigenous people, although the NAO has the highest indigenous population.

### Access to alcohol

There is less access to alcohol in the NAO compared to the AO. Indeed, as the NAO has no permanent road connection with the rest of Russia, goods like alcohol must be ordered outside the region and then transported by plane, winter roads, or ships during the short summer. Despite this, mass-produced alcohol remains the norm in the NAO. Homemade alcohol, known as “moonshine” or “samogon,” is not common as the ingredients required to make it, such as sugar, are too expensive. Vodka is the most popular type of alcohol in the region, though wine has been gaining favor in recent years. The amount of alcohol sold illegally in the region, be it mass-produced or homemade, remains unknown, but is likely to be high.

Access to alcohol is far less fettered in the AO than in the NAO, as transportation and delivery by road and railway is easy, and distribution by shops is well organized. In the AO, and especially in Arkhangelsk, the drinking culture is like that of other towns in Russia, with bars, pubs, and restaurants that provide alcoholic beverages. However, as in the NAO, the amount of alcohol sold illegally is unknown.

Legislative restrictions for alcohol sales were implemented in Russia in 2011, which prohibited the sale of alcohol between 11:00 p.m. and 8:00 a.m. Each region also has its own restrictions: in the AO, alcohol sales are prohibited between 9:00 p.m. and 10:00 a.m., whereas in the NAO sales of hard liquor and wine are prohibited between 8:00 p.m. and 11:00 a.m., and beer cannot be sold between 11:00 p.m. and 8:00 a.m.

**Fig. 1.**
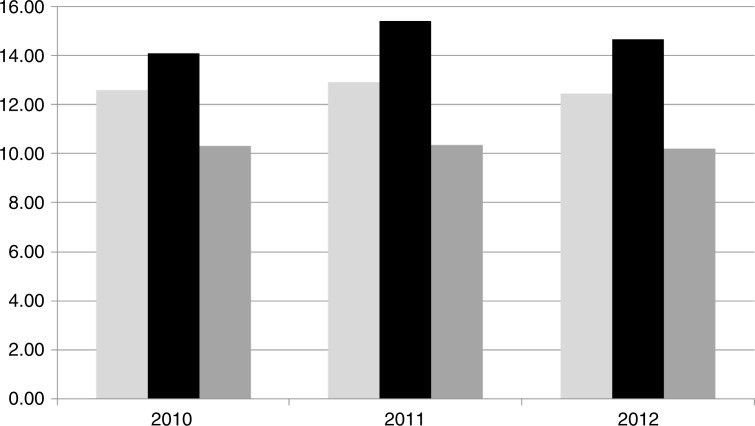
Official alcohol sales in Arkhangelsk Oblast (left), the Nenets Autonomous Okrug (middle), and Russia (right) in 2010–2012 (in liters of pure alcohol per capita divided by the number of individuals over 15 years of age in the corresponding region).

Alcohol consumption in Russia is among the highest in the world (18); consumption is assumed to be higher than figures on alcohol sales indicate (6) due to consumption of illegally sold liquor, homemade liquor and other illegal spirits. The available figures on alcohol sales in the NAO for 2010–2012 were higher than corresponding figures from the AO: 12.05–13.13 and 10.63–11.04 L of pure alcohol per capita, respectively (Rosstat), which is higher than the national average for the same period (Fig. 1). We therefore assume that that alcohol consumption in the NAO is higher than that in the AO and in Russia.

After comparing data from 27 world nations, Lester concluded that alcohol consumption predicted increased suicide rates (20). Several studies have tried to confirm the hypothesis of a direct association between alcohol consumption and suicide in Russia (21, 22). Nemtsov (21) showed how a decrease in per capita alcohol consumption in Russia in 1985–1987, followed by an increase after 1987, was reflected in the decline and increase of national suicide rates in the same periods. Other studies have described a strong correlation between suicide rates and alcohol poisoning rates (23). Razvodovsky reported that an increase in alcohol consumption of 1 L elevated the suicide rates in Russia by 7% in males and by 3.2% in females (24). There is evidence that only restrictions on the availability of alcohol can decrease suicide mortality (4).

### Suicides and presence of alcohol in the blood

National data from different Russian regions (25) have shown a higher proportion of BAC+ suicide cases in males (60.2%) than in females (30%). Our findings from the AO demonstrated the same tendency, with 59.3% BAC+ cases in males and 46.6% BAC+ cases in females. Results from the NAO showed a higher proportion of BAC+ suicides in both genders compared with the AO (74.1% in males and 82.9% in females).

There are no national data available on BAC in suicide victims in Russia, but such data have been reported from other countries. Mean BAC in suicide cases among Native Americans was 1.99‰ in males and 1.80‰ in females (10). Suicide victims in Sweden showed mean BAC of 1.34‰ in males and 1.25‰ in females (11). Corresponding figures from Brazil were 1.84‰ for females and 1.8‰ for males (26).

This study supports the hypothesis that alcohol intake prior to suicide is common in Russia (23) and probably plays an important role in suicide in indigenous territories like the NAO. We found a higher prevalence of BAC+ suicides (both genders) in the NAO than in the AO, and also a higher suicide rate in the NAO than in the AO. This may be seen as the effect of the proximal risk factor (according to Hufford's statement); that is, acute intoxication is an important risk factor for suicidal behavior, as alcohol helps to overcome any inherent resistance to committing suicide (2).

The mean BAC of suicide cases in the NAO and the AO were slightly different both in males and in females. Time elapsed between death and autopsy could be different in the two study cites, representing a potential bias. However, when we calculated this value, we found little difference (5.3 days in NAO and 4.8 in AO), and after discussion with forensic experts, we concluded that this could not explain the difference in mean BAC. Neither did forensic literature support this hypothesis (27–29).

### Alcohol and suicide methods

The most violent suicide methods were hanging in the AO and use of firearms in the NAO. These cases were also most often BAC+. In 1998, Wasserman et al. (30) reported that female suicide and alcohol consumption in the former USSR, as well as violent death and alcohol consumption, were positively correlated. The same study suggested that alcohol had a lower explanatory value in female suicides and female violent deaths compared with male suicides and male violent deaths, due to the lower attributable fraction of alcohol in suicide cases of females (27%) compared to males (50%) (30).

Our results from the NAO show that females more often use alcohol prior to committing suicide, likely to better overcome any resistance to the use of violent suicide methods. The literature shows that females more often use non-violent suicide methods like poisoning (1). Our data from the NAO (14) showed that 80% of suicides by hanging and 100% of suicides by gunshot in indigenous and non-indigenous females took place after alcohol intoxication. Higher BAC in female suicides than in male cases has also been reported in other studies (26). A significantly higher proportion of female suicide cases with a BAC>2.0‰ in the NAO compared to the AO also points to the role of alcohol intoxication for female suicide in this mostly indigenous population.

But why does alcohol play such an essential role in suicides in Russia, and especially among indigenous peoples? In Western countries, mental depression seems to be the main risk factor for suicide, and subjects with depression are treated with anti-depressive medicines. For a long time in Russia, it was not deemed necessary to undergo highly-specialized treatment for mental diseases like depression. Thus these cases were underdiagnosed and undertreated, even more so among indigenous subjects. Depression is not something people talk about; it is seen as a weakness that should be concealed. Instead of medical treatment, those who suffer from depression tend to self-medicate with alcohol. That may explain why so few (especially females) individuals in Russia use overdosing with prescribed medications, as is often seen in the West. In addition, medication is expensive and less available than alcohol, thus alcohol use tends to “mask” the need for more medical treatment. Health workers do not seem to pay enough attention to this phenomenon, leaving the population to use whatever remedies are readily available, namely alcohol.

Finally, our results from the AO drew a general picture of alcohol use prior to suicide that is typical in national suicide statistics. In the NAO, alcohol intoxication was more of a proximal risk factor for suicide, which may support the idea that alcohol plays a leading role in the high suicide rates in the NAO. Suicide prevention activities in the NAO should include a global alcohol policy with appropriate restrictions. Males aged 10–29 years should be the main target group for suicide prevention in the NAO.

### Limitations

No information on mental disease and depression, history of deliberate self-harm, or suicide attempts was available for the cases of suicide included in the present study. Neither was there any information on history of mental disease, especially depression, among the cases. Altogether 24 cases with missing or incomplete values on BAC in NAO and 13 from AO were excluded from further analyses. This probably did not have any substantial impact on the general picture, and thus likely did not influence our results. These missing data were mainly due to the considerable amount of time that had passed between the date of death and the date the bodies were found and identified, conditions that made an exact determination of BAC almost impossible.

The data sample from the AO was not complete for the entire oblast, as some of the smaller districts have their forensic examinations done at local laboratories. All data from the AO in the study were based on examinations performed at the main regional forensic center in the city of Arkhangelsk. Nevertheless, we do not think that the mentioned conditions impacted our results in any substantial way.

### Strengths

The strength of our study is based on the completeness of information collected by forensic experts in autopsy reports. The same methods and procedures were used to determine and measure BAC in both forensic centers. All data used in the paper were checked and double-checked by health workers at the site and by the authors. The forensic work was very well organized in both the NAO and the AO. Planes and helicopters were used when necessary to transport forensic experts to the sites of suicide investigations. The collection of data for all suicides in both regions, as well as complete autopsy data including BAC, gave us the unique opportunity to compare alcohol in the suicide cases of two neighboring regions in the northwest of Russia, the one populated with almost 100% ethnic Russians (AO), the other with a considerable proportion of indigenous peoples (NAO).

### Validity and completeness of data

The police and the Investigation Committee must investigate all suicides in Russia to identify potential criminal cases. A forensic examination immediately follows the primary police investigation. In few cases in males and females, BAC was not analyzed, most often due to a state of extreme decay of the human remains.

## Conclusion

Variations in BAC among suicide victims in the NAO and the AO may reflect different relationships between suicide and alcohol in these two regions. Alcohol was more often observed in the blood of suicide cases in the NAO (both genders) than in the neighboring Russian region of AO. This factor may influence the higher suicide rates in the NAO.
